# Insight into the microbial world of *Bemisia tabaci* cryptic species complex and its relationships with its host

**DOI:** 10.1038/s41598-019-42793-8

**Published:** 2019-04-25

**Authors:** Hua-Ling Wang, Teng Lei, Wen-Qiang Xia, Stephen L. Cameron, Yin-Quan Liu, Zhen Zhang, Maruthi M. N. Gowda, Paul De Barro, Jesús Navas-Castillo, Christopher A. Omongo, Hélène Delatte, Kyeong-Yeoll Lee, Mitulkumar V. Patel, Renate Krause-Sakate, James Ng, San-Ling Wu, Elvira Fiallo-Olivé, Shu-Sheng Liu, John Colvin, Xiao-Wei Wang

**Affiliations:** 1 Institute of Insect Sciences, Zhejiang University, 866 Yuhangtang Road, Hangzhou, 310058 China; 2 Natural Resources Institute, University of Greenwich, Kent, ME4 4TB United Kingdom; 3 Department of Entomology, Purdue University, 901West State Street, West Lafayette, IN 479074 USA; 4 CSIRO Ecosystem Sciences, Brisbane, QLD 4001 Australia; 5 Instituto de Hortofruticultura Subtropical y Mediterránea “La Mayora”, Universidad de Málaga – Consejo Superior de Investigaciones Científicas (IHSM-UMA-CSIC), 29750 Algarrobo-Costa Málaga, Spain; 6 National Crops Resources Research Institute, Namulonge, P.O. Box, 7084 Kampala, Uganda; 7 CIRAD, UMR PVBMT CIRAD-Universitéde La Réunion, Pôle de Protection des Plantes, 7 chemin de l’IRAT, 97410 Saint-Pierre Ile de La Réunion, France; 8 School of Applied Biosciences, Kyungpook National University, Daegu, 702-701 Republic of Korea; 9 UNESP, Faculdade de Ciências Agronômicas, 18610-307 Botucatu, Brazil; 10 Department of Plant Pathology and Microbiology, University of California, Riverside, California, 92521 USA

**Keywords:** Taxonomy, Entomology

## Abstract

The 37 currently recognized *Bemisia tabaci* cryptic species are economically important species and contain both primary and secondary endosymbionts, but their diversity has never been mapped systematically across the group. To achieve this, PacBio sequencing of full-length bacterial 16S rRNA gene amplicons was carried out on 21 globally collected species in the *B. tabaci* complex, and two samples from *B. afer* were used here as outgroups. The microbial diversity was first explored across the major lineages of the whole group and 15 new putative bacterial sequences were observed. Extensive comparison of our results with previous endosymbiont diversity surveys which used PCR or multiplex 454 pyrosequencing platforms showed that the bacterial diversity was underestimated. To validate these new putative bacteria, one of them (*Halomonas*) was first confirmed to be present in MED *B. tabaci* using Hiseq2500 and FISH technologies. These results confirmed PacBio is a reliable and informative venue to reveal the bacterial diversity of insects. In addition, many new secondary endosymbiotic strains of *Rickettsia* and *Arsenophonus* were found, increasing the known diversity in these groups. For the previously described primary endosymbionts, one *Portiera* Operational Taxonomic Units (OTU) was shared by all *B. tabaci* species. The congruence of the *B. tabaci*-host and *Portiera* phylogenetic trees provides strong support for the hypothesis that primary endosymbionts co-speciated with their hosts. Likewise, a comparison of bacterial alpha diversities, Principal Coordinate Analysis, indistinct endosymbiotic communities harbored by different species and the co-divergence analyses suggest a lack of association between overall microbial diversity with cryptic species, further indicate that the secondary endosymbiont-mediated speciation is unlikely to have occurred in the *B. tabaci* species group.

## Introduction

Insects’ bacterial endosymbionts play a critical role in supplementing the diet of their hosts, particularly for those on nutritionally simple diets such as cellulose or plant-phloem sap^1^. Endosymbionts are classified into primary (P-endosymbionts) and secondary symbionts (S-endosymbionts). The major role of the former is to provide essential amino acids, vitamins and nutrients missing from the insect host’s diet. *Buchnera aphidicola*^2,3^ and *Wigglesworthia glossinidia*^4^, for example, complement the diet and metabolic capacity of aphids and tsetse flies respectively. In contrast, the roles of secondary endosymbionts are related to host adaptation, especially aspects of host survival, competitive capacity and providing resistance against natural enemies and pesticides^5–7^. Therefore, P-endosymbionts are generally always presented in a host species, while the prevalence of S-endosymbionts varies between populations within a species.

The whitefly, *Bemisia tabaci* (*sensu* Russell) (Hemiptera: Sternorrhyncha: Aleyrodidae) is a global agricultural pest and a species complex that comprises more than 37 cryptic species (as defined by species delimitation metrics)^8,9^. *B. tabaci* has both P- and seven genera of S-endosymbionts which have been recorded from the different member species in the previous 15 years: *Hamiltonella*^10^, *Arsenophonus*^10^, *Cardinium*^11^, *Wolbachia*^12^, *Fritschea*^13^, *Rickettsia*^14^ and *Hemipteriphilus*^15^. The diversity of S-endosymbionts, however, has not previously been mapped systematically across the entire *B. tabaci* group. Thus, the distributions of S-endosymbionts both at the cryptic species level and across evolutionary lineages within *B. tabaci* were unknown.

Many studies have surveyed the S-endosymbionts using 16S ribosomal RNA (rRNA) molecular markers by normal PCR in field-collected whiteflies^16–18^ and showed that the infection incidences of different S-endosymbionts differ. But most of these studies are mainly focused on the diversity of endosymbionts in *B. tabaci* with restricted region distributions and lack an angle of linking these diversities with its hosts. Also, a major limitation of these studies is the unreliability of the diagnostic PCR techniques used. Ji, *et al*.^17^, for example, found higher incidence of *Wolbachia* infections using nested-PCR than in previous studies using universal primers in end-point PCR^16,17^. Primers developed for *Arsenophonus* were also found to amplify *Rickettsia*^18^ thus making the currently available methods for S-endosymbionts diagnosis and diversity unreliable. We considered, therefore, that an accurate understanding of *B. tabaci* endosymbionts required both systematic sampling and robust evaluation with high-efficiency detection methods. Over the past 10 years, advances in sequencing technologies have enabled considerable progress in the field of microbial ecology and revolutionized the characterization of complex microbial communities^19^. In particular, one of the third generation sequencing technologies, the PacBio Single Molecule, Real-Time (SMRT) DNA sequencing platform has demonstrated that it can generate high quality, full-length reference sequences of 16SrRNA genes, provide a reliable adjunct, and enable more accurate phylogenetic resolution of microbial communities^20^. In this study, therefore, we chose the PacBio sequencing instrumentation for the first time to systematically study the endosymbionts diversity of *B. tabaci* cryptic species.

Aside from the complicated distribution patterns of the S-endosymbionts, all *B. tabaci* species also contain the P-endosymbiont (*Candidatus* Portiera aleyrodidarumin) bacteriocytes^21^, which is transmitted vertically from mother to offspring and have been shown to have evolved in parallel with their insect hosts for millions of years^22^. As a member of sap-feeding insects (suborder Sternorrhyncha), the characterization of this primary endosymbiont associated with its host always indicate coevolution between P-endosymbionts and their hosts, which have been observed in many cases, such as (i) between *Portiera* and whiteflies^23,24^, (ii) *Buchnera aphidicola* and aphids^25,26^, (iii) *Carsonella ruddii* and psyllids^27,28^, as well as (iv) *Moranella endobia* and *Tremblaya princeps* in mealybugs^29^. Co-evolution between *B. tabaci* complex species and *Portiera*, however, has not previously been studied systematically across the group.

The aims of this study, therefore, were to: (i) investigate whether PacBio SMRT technology can improve detection and accurate identification of the known and unknown symbionts in the *B. tabaci* species group; (ii) evaluate bacterial diversity in the *B. tabaci* species complex; (iii) determine co-phylogenetic patterns between P-endosymbionts and *B. tabaci* species on a global scale and better understand the evolutionary pattern(s) of P-endosymbionts and *B. tabaci*.

## Results

### PacBio DNA sequencing of bacterial community in the *B. tabaci* species

**Figure 1:**
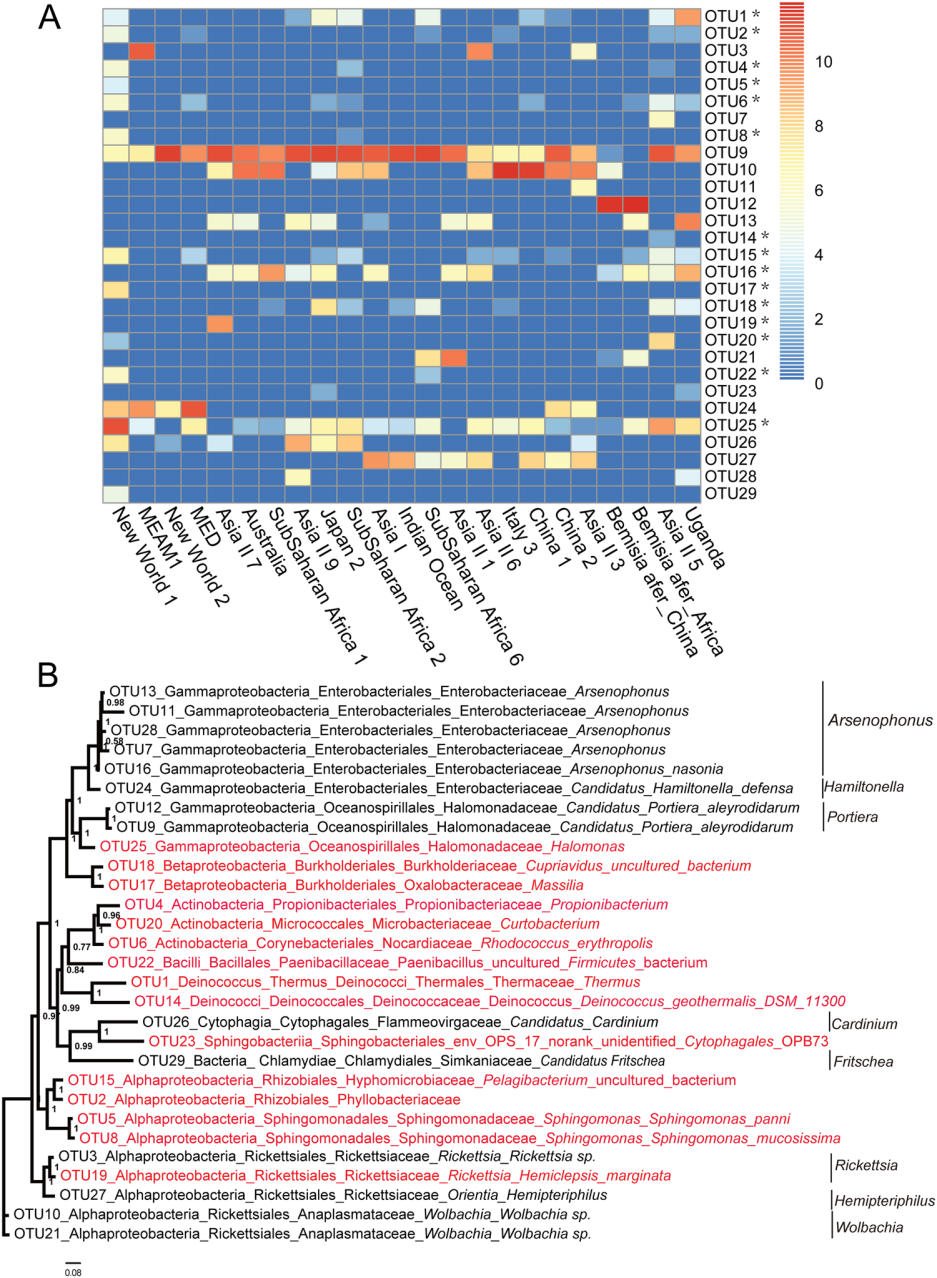
OTU profiles of the samples and phylogenetic analysis of all OTUs. **(A)** Heat map of OTU profiles (y-axis) inferred from PacBio. The x-axis shows 23 samples. The number of reads per OTU was normalized by total number of reads from the sample. For each sample, the number of reads per OTU represents total number of reads (the number of reads were transformed to log 2) from the sample. The asterisk denoted OTUs show the new OTUs found in this study. (**B**) The Bayesian unrooted phylogenetic tree of representative sequences OTUs. Red text labels represent the new OTUs. The scale bar at the base indicates number of substitutions per site.

To accurately estimate the bacteria diversity across evolutionary lineages within *B. tabaci*, a reliable and promising PacBio DNA platform was applied to generate 16S rRNA sequences of 1,513-bp from 21 *B. tabaci* and two outgroup species of *B. afer* with collection information in Table 1. In summary, we obtained a total 70,938 high-quality reads, and the reads count per sample ranged from 2,123–4,184 (Table S1). After quality filtering and removal of chimeric sequences, 65,774 clean 16S rRNA reads were remained and clustered into 29 OTUs using ≥97% sequence identity as the cutoff (Fig. 1A), of which 28 were found in *B. tabaci* species. The alpha diversities for bacterial OTUs from different cryptic species ranged from 0.21–1.59 Shannon index and 0.27–0.97 Simpson index, which are low (Table 2) yet similar to the result obtained by Jing, *et al*.^30^. In addition, the most diverse bacterial OTUs sets were detected in Asia II 6, New World 1, Asia II 3, Asia II 5, SubSaharan Africa 1 and Asia I. Based on the rarefaction curve for every sample with a characterization of tending to saturation (Fig. S1A, Supporting information), we deduced that the OTUs detected were representative of the 16S amplicons in each sample. However, due to under-sampling of field population diversity, there are likely to be additional bacteria yet to be found in *B. tabaci* species (Fig. S1B, Supporting information).

**Table 1:** Provenance information of the *B. tabaci* species used in this study.

Cryptic species	Location	Host plant	GenBank accession number of 16S	GenBank accession number of RER region
Asia I	India	*Gossypium* sp.(cotton)	MG837029	MG063856
Asia II 1	Jiande, Hangzhou, Zhejiang, China	*Gossypium* sp.(cotton)	MG837043	MG063871
Asia II 3	Yuhang, Hangzhou, Zhejiang, China	*Glycine max* (soybean)	MG837051	MG063860
Asia II 5	India	*Manihot esculenta* (cassava)	MG837042	MG063870
Asia II 6	Baise, Guangxi, China	*Ipomoea batatas* (sweet potato)	MG837033	MG063861
Asia II 7	Guangzhou, Guangdong, China	*Codiaeum variegatum* (variegated laurel)	MG837045	MG063873
Asia II 9	Shaoyang, Hunan, China	*Ipomoea batatas* (sweet potato)	MG837044	MG063872
Australia	Bargara, Queensland	*Euphorbia cyathophora*	MG837046	MG063874
Australia_E	Kununurra, Western Australia	*Emilia sonchifolia*	MG837030	MG063857
*Bemisia afer*_Africa	Entebbe, Uganda	*Manihot esculenta* (cassava)		
*Bemisia afer*_China	LinYi, China	*Chrysanthemun coronarium*		
China 1	Yuhang, Hangzhou, Zhejiang	*Ipomoea batatas* (sweet potato)	MG837031	MG063858
China 2	Gaoyao, Zhaoqing, Guangdong, China	*Cucurbiat moschata* (pumpkin)	MG837032	MG063859
Indian Ocean	Saint Pierre la Réunion	*Gossypium* sp. (cotton)	MG837038	MG063866
Italy 3	Spain	*Dorycnium rectum*	MG837041	MG063869
Japan 2	Korea	*Lonicera japonica* (honeysuckle)	MG837052	MG063877
Mediterranean	Ningbo, Zhejiang, China	Capsicum annuum (pepper)	MG837040	MG063868
Mediterranean_Uganda	Uganda	*Ipomoea batatas* (sweet potato)	MG837039	MG063867
Middle East-Asia Minor 1	Wenzhou, Zhejiang, China	*Solanum melongena* (eggplant)	MG837037	MG063865
New World 1	unknown	Unknown	MG837049	MG063878
New World 2	Brazil	*Euphorbia heterophylla*	MG837048	MG063876
SubSaharan Africa 1	Africa	*Manihot esculenta* (cassava)	MG837035	MG063863
SubSaharan Africa 2	Spain	*Ipomoea indica*	MG837034	MG063862
Uganda	Uganda	*Ipomoea batatas* (sweet potato)	MG837047	MG063875
SubSaharan Africa 6	Uganda	*Mentha* (mint)	MG837036	MG063864

**Table 2:** Diversity indices of full-length 16S rRNA gene amplicons from 23 *B. tabaci* samples.

Sample ID	Reads	OTU*	Shannon (97%)	Simpson (97%)
Asia I	2929	8	1.0300 (1.0000,1.0600)	0.4342 (0.4198,0.4486)
Asia II 1	2602	5	0.9200 (0.8900,0.9500)	0.4517 (0.4431,0.4603)
Asia II 3	1921	8	1.3400 (1.3000,1.3800)	0.3483 (0.3311,0.3656)
Asia II 5	2953	13	1.1200 (1.0800,1.1600)	0.4601 (0.4429,0.4774)
Asia II 6	2018	13	1.5900 (1.5500,1.6300)	0.2748 (0.2597,0.2898)
Asia II 7	3139	7	0.8600 (0.8300,0.8900)	0.5325 (0.5165,0.5485)
Asia II 9	2724	9	0.8000 (0.7700,0.8400)	0.5948 (0.5749,0.6147)
Australia	2514	6	0.8500 (0.8200,0.8700)	0.4671 (0.4605,0.4737)
China 1	3149	9	0.5700 (0.5400,0.6100)	0.7379 (0.7184,0.7574)
China 2	3029	7	0.9700 (0.9400,1.0000)	0.4524 (0.4396,0.4653)
Indian Ocean	2725	4	0.5100 (0.4900,0.5400)	0.6890 (0.6705,0.7074)
Italy 3	3182	8	0.2100 (0.1800,0.2400)	0.9190 (0.9061,0.9320)
Japan 2	3006	11	0.7800 (0.7300,0.8300)	0.6856 (0.6642,0.7069)
Mediterranean	2820	6	0.7900 (0.7700,0.8200)	0.5251 (0.5106,0.5397)
Middle East-Asia Minor 1	2710	7	0.8100 (0.7800,0.8400)	0.5191 (0.5041,0.5341)
New World 1	3134	15	1.4300 (1.3800,1.4800)	0.4330 (0.4133,0.4527)
New World 2	2696	3	0.1900 (0.1700,0.2200)	0.9114 (0.8971,0.9258)
SubSaharan Africa 1	2827	5	1.0900 (1.0700,1.1000)	0.3486 (0.3431,0.3541)
SubSaharan Africa 2	3062	13	0.9700 (0.9300,1.0100)	0.5316 (0.5119,0.5512)
SubSaharan Africa 6	2742	10	0.5000 (0.4600,0.5400)	0.7907 (0.7709,0.8106)
Uganda	3009	13	1.6000 (1.5800,1.6300)	0.2277 (0.2221,0.2333)
*Bemisia afer*_Africa	3683	10	0.3100 (0.2800,0.3400)	0.8870 (0.8730,0.9010)
*Bemisia afer*_China	3200	7	0.0900 (0.0700,0.1100)	0.9722 (0.9642,0.9801)

*Operational taxonomic units.

### The bacterial community of *B. tabaci*

**Figure 2:**
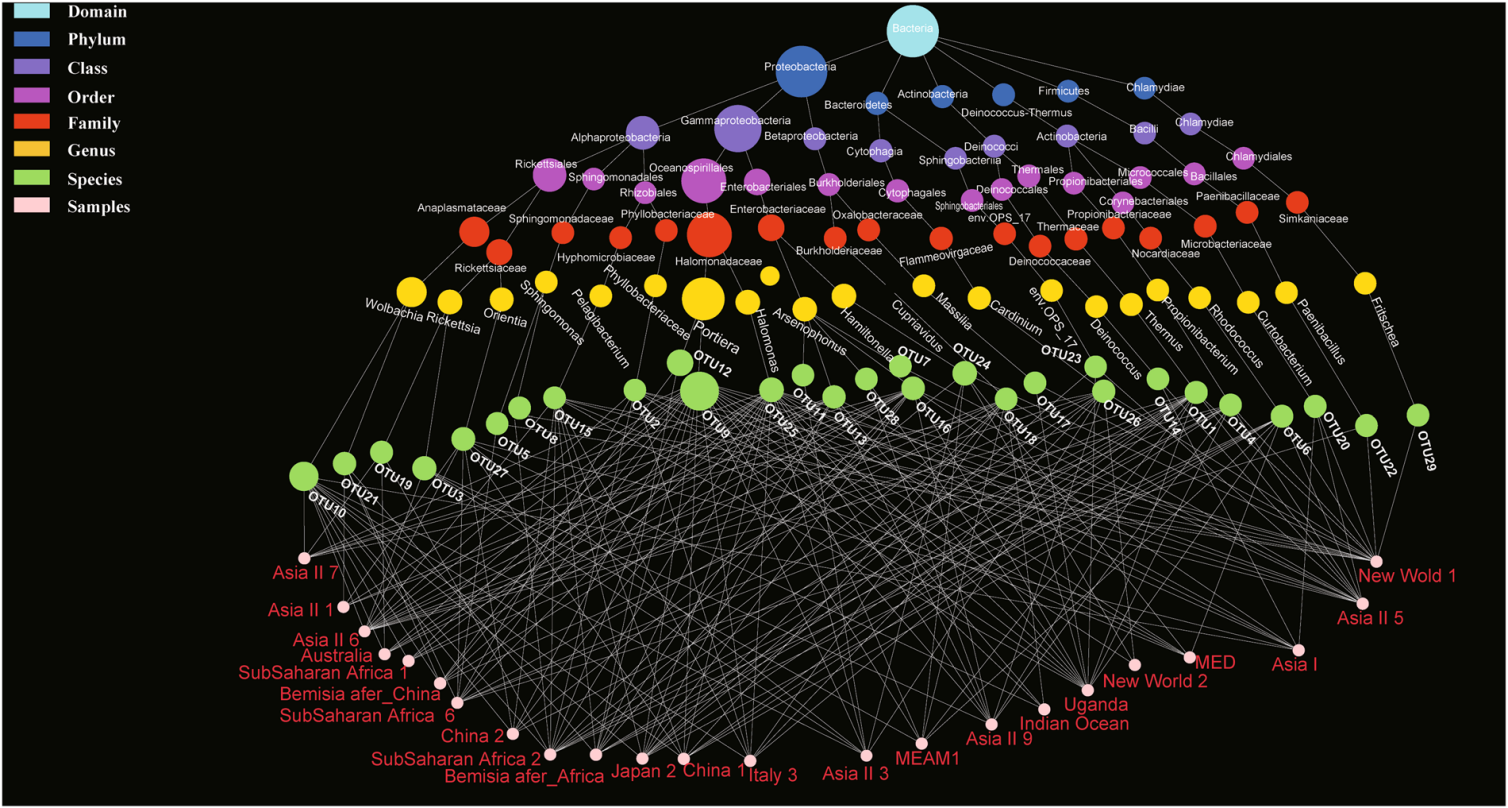
Bacteria composition showed in tested samples. Different bubble colors represent different taxonomic levels, while the different bubble sizes represent the relative number of different bacteria. The species names of OTUs are in Fig. 1B.

The 29 bacterial OTUs represent a total of 21 genera, 18 families, 15 orders and nine classes (Fig. 1) and the distributions across the different cryptic species are shown in Fig. 2. As expected, and importantly, our findings included all previously reported endosymbionts of *Bemisia* (P-endosymbiont: *Portiera*; and seven S-symbionts: *Arsenophonus*, *Cardinium*, *Fritschea*, *Hamiltonella*, *Hemipteriphilus*, *Rickettsia* and *Wolbachia*) (Fig. 1A) which were representatives of the classes of Gamma-proteobacteria, Chlamydiae, Cytophagia and Alpha-proteobacteria (Fig. 1B).

Other than *Porteria*, which was present in every species, the bacteria diversity in various whitefly cryptic species differed substantially. At higher taxonomic levels, the community was dominated by Gamma-proteobacteria and Alpha-proteobacteria. At the ordinal level, Oceanospirillales was the most abundant, as this includes the P-endosymbiont, which was present in all *B. tabaci* species. PCoA based on weighted and unweighted UniFrac distances did not show any significant differences in proportions of bacteria among the samples (Fig. S2, Supporting information).

Several other bacteria not previously reported as endosymbionts of *Bemisia* were found (Fig. 1B, red colored species), such as *Halomonas* (also reported in the gut of many insects), *Massilia*, *Pelagibacterium* and *Curtobacterium*. The distribution of taxa among the *B. tabaci* examined was relatively non-specific (Fig. 2).

### Validation of a new micro-organism in *B. tabaci* by next-generation sequencing and FISH

**Figure 3:**
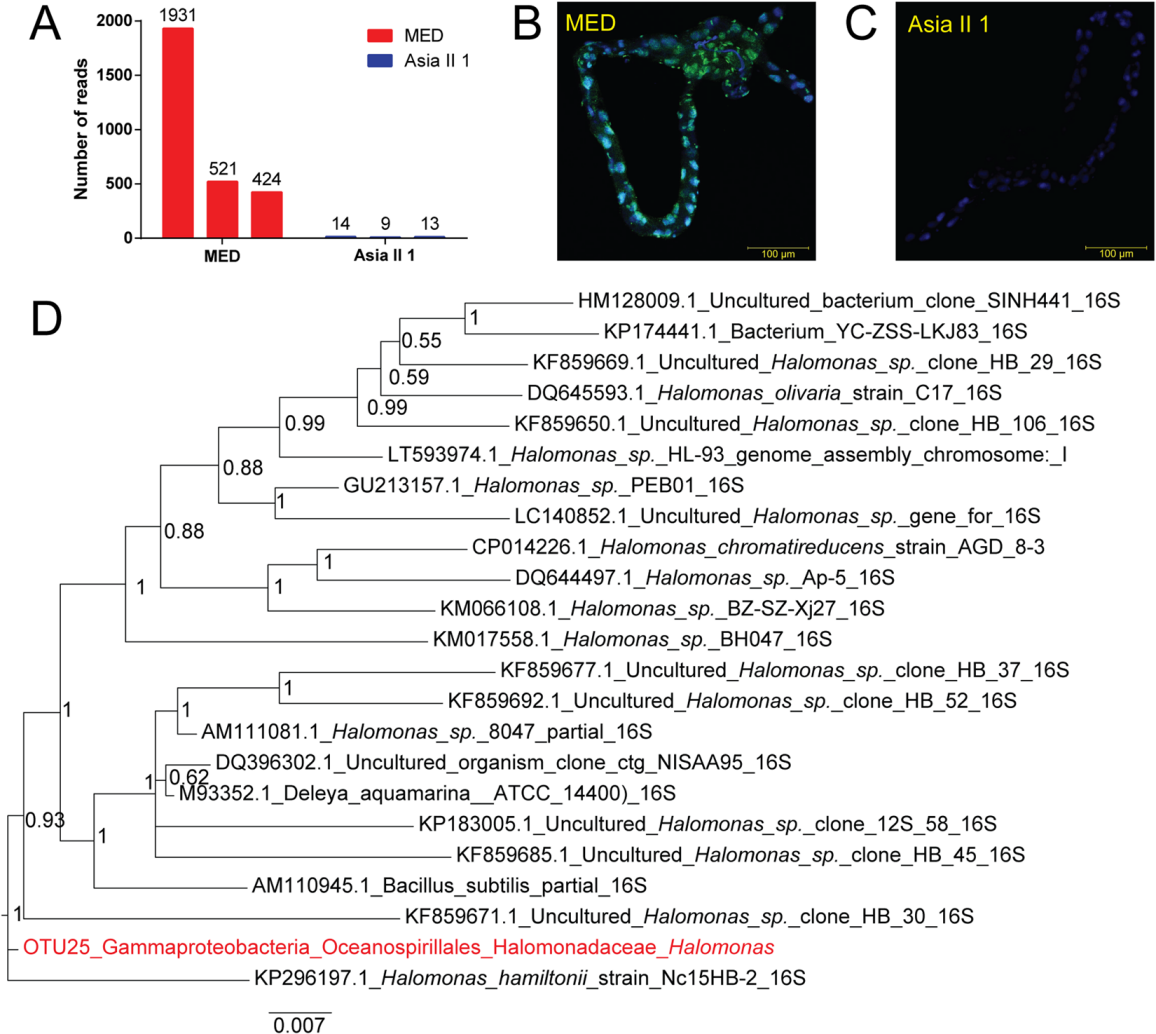
*Halomonas* appeared in the midgut of MED *B. tabaci* verified by application of the v4-v5 region of using dissected midguts **(A)** and fluorescence *in situ* hybridization **(B,C)**. **(D)** Phylogenetic tree revealed the relationship of this *Halomonas* strain with the other published ones. New *bacteria*-specific probes (green) conjugated to cy5 were used, and the nuclei were stained with DAPI (blue). *Portiera* is used as a control for *Halomonas*. *Portiera*-specific probe (red) is expected not to have occurred in the gut of *B. tabaci*. B, *Halomonas* was present in gut of MED; C, *Halomonas* was not detected in the gut of Asia II 1 due to its low content.

One of the novel bacteria *Halomonas* sp. has been reported previously from the gut of other insects, and so, to detect the reliability of the PacBio sequencing platform, through feasibility evaluation, this strain was chosen to determine whether it was also distributed in the midgut of *B. tabaci*. Two subsequent experiments of illumina midguts -target sequencing and FISH were conducted. MED and Asia II 1 species are picked as midgut resources, as it to be widely distributed in the MED *B. tabaci* species and extremely lower in Asia II 1 species. Illumina midguts-target sequencing results showed that *Halomonas* was clearly presented in the MED midguts (reads number: 1931, 521 and 424) in all the post-emergence time periods (Fig. 3A). Identically, the specific designed FISH probe showed it was abundant and distributed in the MED *B. tabaci* midgut (Fig. 3B). As a negative control, it was not apparent in the Asia II 1 midgut (Fig. 3C). At the last, to give a general idea of the relationships of this strain with the other finding species under the same genus, the positions of this *Halomonas* bacteria strain (OTU 25) amongst the published *Halomonas* strains was then deduced by including 1,529-bp 16S in a phylogenetic analysis (Fig. 3D).

### Distribution of the well-known endosymbionts in *B. tabaci*

**Figure 4:**
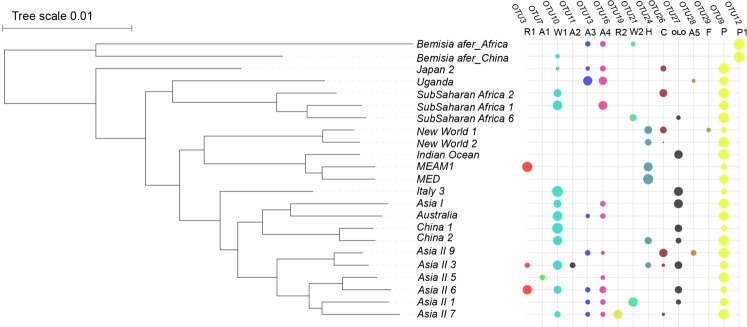
Relative abundance (the number of reads were transformed to log 2 and then multiplied by 10) of PacBio reads assigned to bacterial symbionts of *B. tabaci* cryptic species. R, A, W, H, C, OLO, F and P represent *Rickettsia*, *Arsenophonus*, *Wolbachia*, *Hamiltonella*, *Cardinium*, *Hemipteriphilus*, *Fritschea* and *Portiera*, respectively. The R1 have high sequence identity with *Rickettsia* described by Gottlieb *et al*., (2006). The W1 have a high sequence identity with *Wolbachia* described by Bing *et al*., (2014). Bubble sizes indicate read abundance of individual bacteria in each sample (see scale), with alternate colors to facilitate discrimination between rows; operational taxonomic units (OTUs) less than 10 reads were not included (the full list of OTUs is provided in Table S2).

To present the well-known endosymbionts profile in *B. tabaci*, the distribution of the eight previously reported endosymbionts amongst *B. tabaci* cryptic species is mapped against a phylogeny of host mitochondrial relationships (Fig. 4). Clearly, two different P-endosymbiont *Portiera* OTUs were found with one in all *B. tabaci* species (OTU9) and a second one in *B. afer* (OTU12). Of the seven S-endosymbionts, a total of five *Arsenophonus* OTUs were found in this study: OTU7 (A1), OTU11 (A2), OTU13 (A3), OTU16 (A4) and OTU28 (A5). Of these, OTU16 (A4) has more than 97% nucleotide identity to *Arsenophonus*_*nasoniae* (son-killer_infecting_*Nasonia_vitripennis*) and was detected in 11 different cryptic species of *B. tabaci* plus both *B. afer* samples. Only one *Cardinium* OTU (OTU26) was detected, occurring in seven *B. tabaci* cryptic species. One *Fritschea* OTU (OTU29) was found, restricted to New World 1^13^. One *Hamiltonella* OTU (OTU24) was found, occurring in six *B. tabaci* cryptic species. Singh, *et al*.^31^ and Bing, *et al*.^15^ reported *Hemipteriphilus/OLO*(OTU27) from specimens of China 1 (collected from China) and Asia I and Asia II 1, both collected from north India. However, OTU27 was found in Asia II 3, Asia II 6, China 1, China 2, Indian Ocean and SubSaharan Africa 6 in our study. Two OTUs were detected from each of the two remaining S-endosymbionts: *Rickettsia* (OTU3 and OTU19) and *Wolbachia* (OTU10 and OTU21).

**Figure 5:**
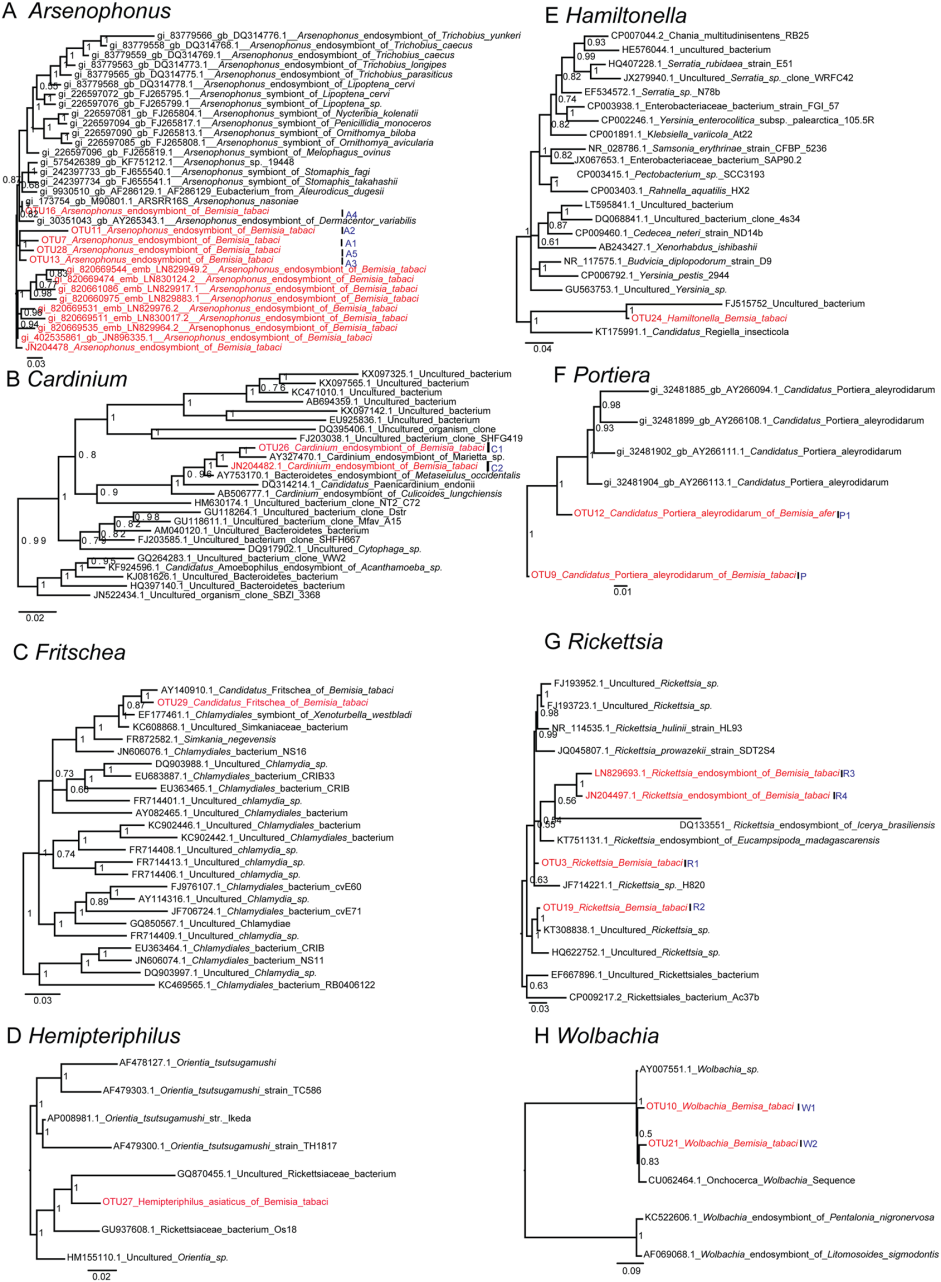
All known symbionts found in the *B. tabaci* cryptic species complex in this study combined with those available from Genbank (**A**–**H**). P represents the primary endosymbiont discovered from *B. tabaci*, while P1 represents the primary endosymbiont discovered from *B. afer*. The 16S rRNA lengths used to build the phylogenetic tree for A-H are 1,085 bp, 1,569 bp, 1,194 bp, 1,273 bp, 1,495 bp, 1,433 bp, 1,235 bp, 1,621 bp, respectively. The phylogenetic trees were conducted by Bayesian inference and the posterior probabilities were shown in the trees.

Through comparing our data to that of previous studies (Table S3), although the strains from different studies are incomparable, a relatively higher strain diversity was reflected by PacBio and several newly discovered symbionts (OTU7, OTU11, OTU3, OTU28 and OTU19) were detected. To improve the systematic knowledge of symbionts in the *B. tabaci* group, we downloaded all 16S rRNA sequences from each symbiont genus and retained one unique sequence as a representative of each 97% identical cluster. Together with the 16S rRNA sequences detected from this study, they were all subjected to phylogenetic analyses, and the results clearly present all strains found for each genus so far in *B. tabaci*. Currently, one *Porteria*, two *Cardinium*, one *Fritschea*, one *Hemipteriphilus*, one *Hamiltonella*, 14 *Arsenophonus*, four *Rickettsia* and two *Wolbachia* species have been reported from *B. tabaci* (Fig. 5).

### Co-evolution analysis of P-endosymbionts and *B. tabaci* hosts

According to previous studies, it has been hypothesized that P-endosymbionts co-evolve with their hosts, while S-endosymbionts do not^23,32^. For instance, there is absence of evidence to support this hypothesis in *B. tabaci*. To achieve this, the most popular and frequently used marker 16S rRNA for bacterial phylogeny and reconstruction of insect-symbiont coevolution^23,29^ were cloned from 23 cryptic species, and subsequently used for generating the phylogenetics of P-endosymbionts. In contrast, full mitogenomes markers were applied to construct phylogenetic topology of host *B. tabaci*.

Unfortunately, preliminary analysis of the 16S rRNA phylogeny lacked sufficient variation for OTU members from different cryptic species for resolution of evolutionary patterns (Fig. S3). To overcome this limitation and find an alternative marker, we looked for markers with sufficient sequence divergence through scanning the three available P-endosymbiont genomes from MEAM1, MED and Asia II 1 *B. tabaci* (“data under submission, DanTong Zhu” for the corresponding author of that study). Briefly, a sliding window approach was applied to determine the local identities of the three well-aligned *Portiera* genomes using a custom Perl script (available from the senior author on request). The well-aligned regions with relative high divergence among the three *Portiera* genomes were selected for further analyses. Totally, five regions with a relatively high evolutionary rate (RER), which were located between non-coding regions, were picked to generate PCR clones from each cryptic species. Eventually, one specific region (PCR primers: F (5′-CACTTGGCGGTGAGGT-3′) and R (5′-ACAATCTTCCATTCTTTCCA-3′)), defined as RER locus with 814 bp, was then chosen to address this question. Filtering process was showed in Fig. S4.

**Figure 6:**
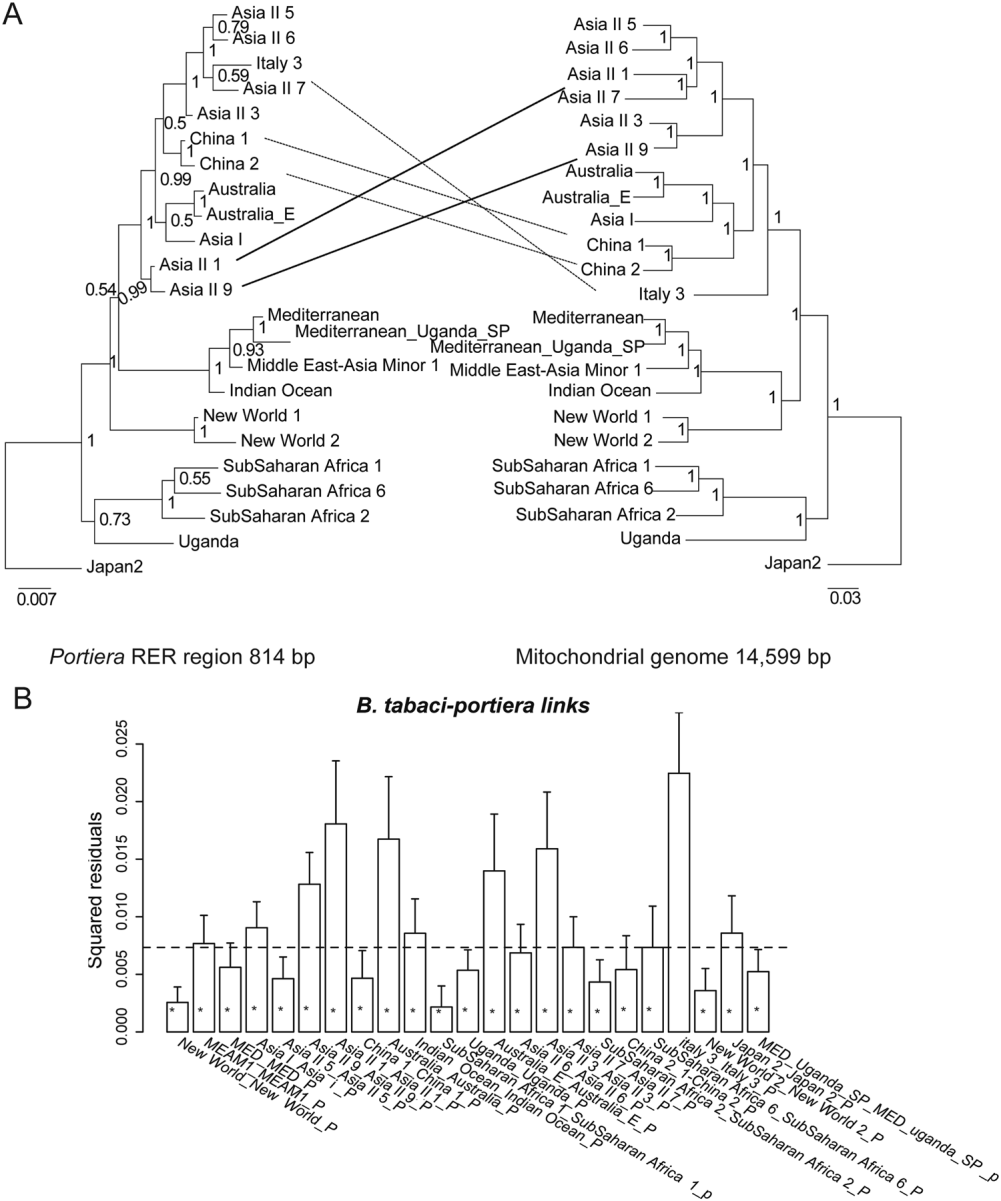
(**A**) Phylogenetic tree from MrBayes analyses of whitefly P-endosymbiont-combined specific DNA nucleotide sequences (left) and host mitochondrial genomes (right). Designations refer to *B. tabaci* species. (**B**) Jacknifed squared residuals (bars) and upper 95% confidence intervals (error bars) associated with each *B. tabaci*–*Portiera* link. PACo was applied to HKY85 genetic distances. The dashed line indicates the median squared residual value. Asterisks identify links significantly supported by ParaFitLink1&2 (a < 0.05).

For the phylogenetic topologies generated from *B. tabaci* and P-endosymbiont, MrBayes and RAxML were applied to analyze the phylogenetic relationships of the *B. tabaci* species using the 14,599-bp mitochondrial sequences and of the P-endosymbiont from the 814 bp RER. Aside from a few branches with low support, the backbone topologies of the BI and ML trees were identical. Thus, only the BI tree is presented here and is used for further cophylogenetic analyses. High congruence between *B. tabaci* mitochondrial and *Portiera* RER phylogenies was presented (Fig. 6A). Similarly, both ParaFit and PACoanalyses provided evidence for significant co-divergence (ParaFitGlobal = 0.0021, *P* ≤ 0.001; a residual sum of squares (m^2^) = 0.1301, *P* = 0.000). Of the 23 host–endosymbiont associations, 21 were significant based on a ParaFit1 value of *P* ≤ 0.05, while 22 were significant based on a Parafit 2 value of *P* ≤ 0.05 (Fig. 6B). Overall, we here demonstrated that P-endosymbionts co-evolve with their *B. tabaci* hosts for the first time.

## Discussion

By using PacBio sequencing platform, 28 bacterial OTUs were discovered in the *B. tabaci* cryptic species complex for the first time, which enriches the bacterial reference database of whiteflies. In comparison, in whitefly research, only the 454 pyrosequencing platform had been used once to investigate the bacterial communities^30^. In that research, fewer than 10 bacterial OTUs were reported through screening representatives of seven cryptic species and three field populations. Apparently, this study adds more samples across the species complex of *B. tabaci* with a promising PacBio technology, which resulted in a reservoir of new information on the bacteria diversity of *B. tabaci*. This study significantly revolutionizes our knowledge on the bacteria diversity of an important economical pest. However, one of the constraints of the PacBio sequencing platform is that relatively high quantities of DNA are needed for library preparation^33^ and it is preferable to carry out PCR-free library preparation to avoid problems generated by amplification. The numbers of adult whitefly individuals we had for each species, however, made it difficult to get enough DNA for PCR-free library construction due to its tiny body size and so we utilized a non-PCR free library construction method to conduct the experiment which is a limitation for this study.

### Novel OTUs found in this study

Fifteen new bacterial OTUs were identified in this study, which hugely increases our catalogue of bacterial diversity in *B. tabaci* species. Some of the new OTUs were close relatives of those from free-living bacteria, and soil or plant associated bacteria, such as *Halomonas*, *Massilia*^34^, *Cupriavidus* (OUT 8, OUT 18)^35^ and *Sphingomonas*^36^, as well as some related to occasional human and plant pathogens such as *Acinetobacter* (OUT 6, OUT 14, OUT 20)^37^ and *Rhizobiaceae*^38^.

Several OTUs, interestingly, could be attributed to known bacterial symbionts from other organisms. OUT 2, a *Phyllobacterium* species (Rhizobiales: Phyllobacteriaceae, Alpha-proteobacteria) has previously been recorded in leaf nodules of a tropical plant species^39^ and was found here in several cryptic species of *B. tabaci*, especially in New World1 (supported by the presence of 27 reads). *Burkholderia* sp. (OUT 17 and OUT 18) that have been reported previously as symbionts of the broad-headed bugs *Riptortus clavatus* and *Leptocorisa chinensis* (Heteroptera: Alydidae)^40^, were found in the New World 1 (OUT 17, 199 reads) and in Japan 2 (OTU 18, 170 reads) species. In addition, *Paenibacillus* (OUT 2, 27 reads) found in the New World is a genus of facultative anaerobic, endospore-forming bacteria that has been reported from a variety of different environments^41,42^.

Gut microbiota are of particular interest, because they are involved in many aspects of microbial pathogen action^43^, such as impacting the development, growth and survival of Hemiptera^44–46^. The gut microbiota of *B. tabaci* has not been investigated in detail—even in the present study, we were only able to show that *Halomonas* sp. was present in the midgut. Such limitations can be attributed to the fact that most of our whitefly samples had been stored in ethanol, from which makes it difficult to cleanly dissect out the midgut. As a first identified midgut associated bacteria in *B. tabaci*, the evidences presented here not only confirmed the reliability of the PacBio result, but also severed as the first steps required in understanding whether it is a gut resident species, as well as the origin and role of this bacterium and its relationship with *B. tabaci* species. Additionally, as Poddar *et al*.,^47^ suggested, *Sphingomonas* is a cultivable gut bacteria across multiple developmental stages of whitefly (Asia II 1 and Asia I). Similarly, *Sphingomonas* was also detected in the New World 1 and New World 2 cryptic species. Unfortunately, the lack of live lab colonies impacts on any further verification of its presence.

### Known symbionts

As a primary endosymbiont, *Portiera* reads were assigned to two different OTUs, one from the 21 *B. tabaci* species and a second from *B. afer*—most probably due to the significant length of time since these clades diverged^48^. The nucleotide identity of the two *Portiera* OTUs representatives was 96%, just outside our range for OTU cutoff (3% divergence). OUT 12 was compared against all *Portiera* 16S rRNA sequences in GenBank, which were all less than or equal to 96% identical. These results suggest that the *Portiera* 16S rRNA might be useful as a distinguishing marker to delineate members of the *B. tabaci* complex from other *Bemisia* species. As such, it shall help draw a clear boundary line between other *Bemisia* clades within the genus.

As regard to the secondary endosymbionts, previously, a total of two *Cardinium* spp.^11,30^, three *Rickettsia* spp. (one unpublished was deposited in the GenBank_LN 8296931)^31,49^ and three *Wolbachia* spp.^12,16,18^ have been discovered in *B. tabaci*. In this study, more novel OTUs were assigned to *Arsenophonus*, *Rickettsia* and *Wolbachia* resulting in at least two *Cardinium* spp.^11,30^, four *Rickettsia* spp.^31,49–51^, three *Wolbachia* spp.^12,16,18,52^, six *Arsenophonus* spp.^10^, one *Hamiltonella*^53^, one *Fritschea*^13^ and one *Hemipteriphilus*^15^ present in the *B. tabaci* species group. This result significantly improves our understanding on the substantial diversity of these endosymbionts. The reasons forthe observed variety and variability of secondary endosymbionts might be as follow: (1) horizontal transmission occurred among phylogenic closely species^54^, (2) horizontal transmission happened through insect parasitoids^55^, (3) as observed for *Rickettsia*, plant-mediated horizontal transmission also could be responsible for other bacteria^56^. Further work is clearly required to examine the above hypothesis and elucidate the mechanisms of transmission and maintenance of this bacterium within and between insects. In addition, due to a range of field populations that have built various, strong associations with different host-plants, field colonies are likely to contain a more varied set of microorganisms than lab strains. In our study, for example, a new *Rickettsia* OTU was detected in the Asia II 7 *B. tabaci*, which was collected from the plant host, *Hibiscus rosa-sinensis* (L.). It is different from the *Rickettsia* OTUs from MEAM1, Asia II 3 and Asia II 6 which were collected from eggplant, cotton and sweet potato, respectively.

### Implications from the overall microbial diversity with host speciation

Apart from the *Portiera* P-endosymbiont, which was presented in all species, there was no significant pattern of S-endosymbiont association with *B. tabaci* species across the group. Multiple comparisons including alpha bacterial diversities, Principal Coordinate Analysis, indistinct endosymbiotic communities harbored by different species and the co-divergence analyses demonstrate the lack of association between overall microbial/S-endosymbiont diversity and cryptic species. Additionally, the majority of newly discovered endosymbionts are mainly secondary endosymbionts, but not the primary endosymbionts. These results indicate that the hypothesis of secondary endosymbiont-mediated speciation is unlikely to have occurred in the *B. tabaci* species group. The primary endosymbiont seems to be more reliable source to study the co-evolutionary speciation. The previous studies of phylogenetic congruence had conducted between distantly related whiteflies (i.e. between genera) and their P-endosymbionts^23^, but no studies had ever performed regard to the co-evolution between a group of closely related cryptic species and its primary endosymbiont, such as *B. tabaci*. In the molecular markers choice, the classical *Portiera* 16S rRNA marker was not able to distinguish P-endosymbionts from different *B. tabaci* cryptic species, which prevented the use of this marker for co-speciation studies. By scanning the three P-endosymbiont genomes generated from three cryptic species, a novel locus was selected to enable the design primers to investigate possible co-divergence of the P-endosymbiont and the host. Finally, significant cospeciating congruence between *B. tabaci* and *Portiera*^57,58^ was confirmed. In all, *Portiera* might have been resident in its host, *B. tabaci* when they were still a single species and subsequently diverged subtly with the divergence of *B. tabaci*. With the developmet of next/third-generation high throughput sequencing to obtain the genomes of P-endosymbionts and *B. tabaci* is more feasible. Further comparison of phylogenomic trees of them with precise fossils and biogeographic time points will allow studying the evolutionary history of their association^24^. Deciphering the evolution of *B. tabaci* and its bacteria might provide far-reaching insights not only on *B. tabaci*-endosymbiont themselves but also on the evolutionary history of other host-parasite pairs. This piece of research, therefore, provides a model framework, tools and methods to further test co-evolution hypothesis for other insect groups and their bacterial associations.

## Conclusion

There are five main conclusions we can draw from our research:

1. Additional bacterial diversity in *B. tabaci*, with 15 new bacteria were found, which increases our understanding of the diversity of symbionts present in *B. tabaci* species and could revolutionize bacteria-insect microbiology, and it is now apparent that the diversity of microorganisms is much greater than previously thought.
2. To detect whether the results reflected by the PacBio are reliable, one of the new micro-organisms (*Halomonas*) was further confirmed to be present in *B. tabaci*. This finding provides robust experimental evidence to credibly support the presence of novel bacteria found in this study.
3. One *Portiera* OTU was present in all *B. tabaci* species, with a second in *B. afer*, indicating *Portiera* 16S might be a highly-effective marker for delineating the *B. tabaci* group members from other whitefly species within the Genus *Bemisia*. Supposing this conclusion is true, it will provide a significant contribution for boosting the taxonomy of some cryptic species groups infected by primary endosymbionts.
4. The hypothesis that P-endosymbionts co-evolve with their cryptic species hosts was firstly confirmed through utilizing a novel developed marker with a more rapid evolutionary rate than the 16S.
5. The lack of association between overall microbial/S-endosymbiont diversity with cryptic species revealed by multiple comparisons (alpha bacterial diversities, Principal Coordinate Analysis, indistinct endosymbiotic communities harbored by different species) indicate that secondary endosymbiont-mediated speciation is unlikely to have occurred in the *B. tabaci* species group.

All in all, these results confirmed that PacBio SMRT technology can improve detection and accurate identification of the known and unknown bacteria in the *B. tabaci* species group. This implies it may be an effective tool for bacteria diversity investigations in other pests. What’s more, as many novel endosymbionts discovered here, the diversity of endosymbionts in the *B. tabaci* (Hemipterans) seems to be much larger than previously thought. This suggests that the endosymbionts diversity in other insects of *Hemipterans* might have been underestimated, and as a consequence, their roles in mediating the host adaptation are largely overlooked. Overall, this study provides important implications for taxonomy, diversity and applied identification of pests known under collective name.

## Material and Methods

### Collection and preservation of specimens

Table 1 lists the meta-data on the *B. tabaci* and *B. afer* specimens, together with available information on host plants, locations, and GenBank information. *B. afer* has a genetically close relationship with *B. tabaci* and provides a good reference to interpret the results. The whiteflies were stored in 100% ethanol at −80 °C until DNA extraction.

### DNA extraction and PCR amplification

To test the purity of each sample, the 5′ partial *mtCOI* gene region for species identification was amplified from *B. tabaci* samples using the new design primer pairs FHL (5′-TGRTTYTTTGGTCATCCVGAAGT-3′) and RHL(5′-TTACTGCACTTTCTGCCACATTAG-3′). DNA was extracted from 10 adult females separately from each sample (whole insect extractions) using the DNeasy animal tissue kit (Qiagen, Germany) following the manufacturer’s protocol and pooled in equal proportions.

### 16S rRNA amplication

The almost full-length, bacterial 16S rRNA genes of 10 individual females mixture of each specimens were amplified by PCR (95 °C for 2 min, followed by 35 cycles at 95 °C for 30 s, 55 °C for 30 s, and 72 °C for 60 s and a final extension at 72 °C for 10 min) using the forward primer 16S-27F (5′-AGAGTTTGATCMTGGCTCAG-3′), and the reverse primer16S-1492R (5′-GGYTACCTTGTTACGACTT-3′) modified from general 16S rRNA gene primers^59^ containing a variable 8 bp barcode sequence. PCR reactions were performed in triplicate, with 20 μL mixture containing 4 μL of 5 × FastPfu Buffer, 2 μL of 2.5mM dNTPs, 0.8 μL of each primer (5 μM), 0.4 μL of FastPfu Polymerase and 10 ng of template DNA. PCR amplicons were extracted from 2% agarose gels and purified using the AxyPrep DNA Gel Extraction Kit (Axygen Biosciences, Union City, CA, USA) according to the manufacturer’s instructions. After that, purified products were quantified by QuantiFluor™-ST (Promega, Madison, WI, USA).

### PacBio 16S library construction and sequencing

Purified PCR products were quantified by Qubit®3.0 (Life Invitrogen) and every 24 amplicons with different barcodes were mixed in equal quantities. Pooled DNA was sequenced using P6-C4 chemistry on a PacBio RS II instrument (Pacific Biosciences, USA) and the entire sequence preprocessing was performed as described by Mosher, *et al*.^60^.

### Processing of sequencing data generated from PacBio platform

For PacBio 16S datasets, raw data processing was carried out using the protocol RS_ReadsOfInsert.1 available in SMRT Portal version 2.7 as described by Hou, *et al*.^61^. Operational Taxonomic Units (OTUs) were clustered with 97% similarity cutoff using UPARSE (version 7.1 http://drive5.com/uparse/), and chimeric sequences were identified and removed using UCHIME. The phylogenetic affiliation of each 16S rRNA gene sequence was analyzed by the tool of RDP Classifier (http://rdp.cme.msu.edu/) against the SILVA^62^ 16S rRNA reference database using a confidence threshold of 70%^63^. To show the distribution of the OTUs along various cryptic species, network analyses was done by Cytoscape^64^.

### Diversity analyses

Rarefaction, Chao, ACE and Shannon diversity indices were performed using a set of Mothur tool^65^. To examine dissimilarities in the composition of bacterial communities, a Principal Coordinate Analysis (PCoA) was performed with an operating principle of using a distance matrix to plot n samples in (n–1)-dimensional space^66^.

### Sequence alignments and phylogenetic tree for each bacterium

Sequences for the toward full-length 16S rRNA, specific region of P-endosymbionts and their corresponding *B. tabaci* hosts were each aligned using default parameters in MUSCLE^67^ as implemented in MEGA 5^68^ or ClustalW-MPI^69^. Alignments were inspected, and ends were trimmed, resulting in 14,599 base pairs (bp) for the *B. tabaci* mitogenome sequences and 795 bp for the *Portiera* genome region.

The 16S rRNA gene data sets of published bacterial communities were downloaded from GenBank for phylogenetic analyses. Multiple 16S rRNA nucleotide sequences were aligned and clustered with a common and an empirical similarity threshold (97%)^70^ by using CD-HIT tool^71^. The Bayesian inference (BI) method was used to build the 16S molecular phylogenetic tree for each bacterium.

### Verification of the presence and distribution of one of the new micro-organisms in the MED *B. tabaci* species

*Halomonas*^72^ has been found in the gut of pine weevils and it was detected in our PacBio data in more than 13 species, including MED, with more than five reads per sample. To determine whether this bacterium was present in the gut of *B. tabaci*, we conducted two subsequent experiments, which were illumina midguts -target sequencing and Fluorescence *in situ* hybridization (FISH) analyses.

Twenty mid-guts of adults were dissected at different times post-emergence (0-day, 0–1 day, 4–5 days) from both lab colony of MED and Asia II 1 (where *Halomonas* reads were absent)^73,74^. DNA was extracted from the midgut and used for sequencing the universal V4-V5 hyper variable region (515F/907R primer set)of 16S rRNA genes^75^ using Illumina Hiseq2500 platform with PE250 mode at Biozeron (Shanghai, China). To aid distinguish samples after sequencing, sample-specific 12-bp barcodes were added to the reverse primers. Different from PacBio sequences processing pipeline, V4-V5 region sequences were analyzed with QIIME software package (Quantitative Insights Into Microbial Ecology) following UPARSE pipeline^76^. OTUs were identified with UCLUST at the 97% sequence similarity level^77^ and a representative sequence from each OTU was aligned using PyNAST^76^.

To visualize the presence or absence of the *Halomonas* in the gut, FISH analyses were performed on dissected midguts as described by Ghanim, *et al*.^78^ with a slight modification. To target the 16S rRNA of the new *Halomonas* micro-organism, the probe Ha-Cy5 (5′-Cy5- TCACCAACTAGCTAATCCGACAT-3′) for the *Halomonas* strain was designed using Primer3 software (http://fokker.wi.mit.edu/primer3/) based on the sequences of the new bacterium strain 16S rRNA. The specificity of the detection was confirmed using the following controls: a no-probe control, an unlabeled competitive suppression control and *Halomonas*-free whiteflies.

### Co-divergence analysis

The global ParaFit^79^ statistic was used to conduct the co-divergence analysis. ParaFit measures two hypotheses: H_0,_ evolution of the hosts and parasites occurred independently; and H_1_, the positions of the individual Host-Parasite (H-P) links are not random but associated with corresponding branches of the two evolutionary trees. A high fit to H_1_ is consistent with cophylogenetic history. In this case, *B. tabaci* represents the host while *Portiera* represents the parasite. They were used to quantify the degree of congruence between *B. tabaci* and P-endosymbiont topologies and identify the individual associations contributing to the co-phylogenetic structure^80^. To get reliable topologies for further analyses, two major phylogenetic methods, BI and Maximum Likelihood (ML) were used to compare phylogenetic structures of P-endosymbiont and *B. tabaci*. BI was conducted using MrBayes 3.2^81^ and ML analyses with RAxMLv 7.0.4, implementing a fast bootstrapping algorithm^82^. Bayesian analysis was conducted in combination with an exact model (GTR + I + G) of molecular evolution, as well as a rapid approximation of a posterior probability tree using Markov Chain Monte Carlo^83^. MrBayes 3.2 was run using four incrementally heated chains and run for 10 million generations, sampling every 5000^th^ generation with four heated chains and a burn-in length of 1 million. To corroborate the phylogenies, as determined through BI, the ML P-endosymbiont and *B. tabaci* phylogenies were generated and the substitution model GTR with CAT approximation was used to incorporate heterogeneity rate across sites. The *B. tabaci* Japan 2 species was chosen as an outgroup owing to this species having a more distant genetic relationship with the other members of the *B. tabaci* species.

Matrices of parasite and host distances were calculated from host and parasite phylogenies with an additional matrix of host–parasite links. Next, ParaFit analyses were performed in R using the package “ape”^84^ with 999 permutations to implement a global test as well as individual links. Each *B. tabaci* species and P-endosymbiont interaction was determined to be significant if either its ParaFitLink1 or ParaFitLink2 *P*-value ≤ 0.05. The recently developed, “Procrustean Approach to Co-phylogeny(PACo)” program^85^, implemented in R using packages ape and vegan^86^, was used to obtain and potentially corroborate, comparable global goodness-of-fit statistics with ParaFit global values. Although some specimens belonged to the same species, each terminal taxon was treated as an independent evolutionary unit as is common in co-speciation literature^87^.

## Supplementary information

Supplementary file

## Data Availability

The raw reads of PacBio sequencing results were deposited in the NCBI Sequence Read Archive (SRA) database (Accession Number: SRP117655).
