# Publisher Correction: Insight into the microbial world of *Bemisia tabaci* cryptic species complex and its relationships with its host

**DOI:** 10.1038/s41598-019-45297-7

**Published:** 2019-06-18

**Authors:** Hua-Ling Wang, Teng Lei, Wen-Qiang Xia, Stephen L. Cameron, Yin-Quan Liu, Zhen Zhang, Maruthi M. N. Gowda, Paul De Barro, Jesús Navas-Castillo, Christopher A. Omongo, Hélène Delatte, Kyeong-Yeoll Lee, Mitulkumar V. Patel, Renate Krause-Sakate, James Ng, San-Ling Wu, Elvira Fiallo-Olivé, Shu-Sheng Liu, John Colvin, Xiao-Wei Wang

**Affiliations:** 1 Institute of Insect Sciences, Zhejiang University, 866 Yuhangtang Road, Hangzhou, 310058 China; 2 Natural Resources Institute, University of Greenwich, Kent, ME4 4TB United Kingdom; 3 Department of Entomology, Purdue University, 901West State Street, West Lafayette, IN 479074 USA; 4 CSIRO Ecosystem Sciences, Brisbane, QLD 4001 Australia; 5 Instituto de Hortofruticultura Subtropical y Mediterránea “La Mayora”, Universidad de Málaga – Consejo Superior de Investigaciones Científicas (IHSM-UMA-CSIC), 29750 Algarrobo-Costa Málaga, Spain; 6 National Crops Resources Research Institute, Namulonge, P.O. Box, 7084 Kampala, Uganda; 7 CIRAD, UMR PVBMT CIRAD-Universitéde La Réunion, Pôle de Protection des Plantes, 7 chemin de l’IRAT, 97410, Saint-Pierre, Ile de La Réunion, France; 8 School of Applied Biosciences, Kyungpook National University, Daegu, 702-701 Republic of Korea; 9 UNESP, Faculdade de Ciências Agronômicas, 18610-307 Botucatu, Brazil; 10 Department of Plant Pathology and Microbiology, University of California, Riverside, California, 92521 USA

Correction to: *Scientific Reports* 10.1038/s41598-019-42793-8, published online 25 April 2019

The original version of this Article contained an error in the author list, where Paul De Barro was erroneously removed during publication. In addition, Elvira Fiallo-Olivé was incorrectly affiliated with ‘National Crops Resources Research Institute, Namulonge, P.O. Box, 7084, Kampala, Uganda’. The correct affiliation is listed below:

Instituto de Hortofruticultura Subtropical y Mediterránea “La Mayora”, Universidad de Málaga – Consejo Superior de Investigaciones Científicas (IHSM-UMA-CSIC), 29750, Algarrobo-Costa, Málaga, Spain

These errors have now been corrected in the PDF and HTML versions of the article.

