# Supplementary material for: Insight into the microbial world of Bemisia tabaci cryptic species complex and its relationships with its host
Source: Sci Rep. 2019 Apr 25;9:6568. doi: 10.1038/s41598-019-42793-8 (PMC6484021; doi:10.1038/s41598-019-42793-8)
Supplement: Supplementary file 1 — Supplementary file [file 41598_2019_42793_MOESM1_ESM.pdf]

Supplementary information for "Insight into the microbial world of *Bemisia tabaci* cryptic species complex and its relationships with its host"

Hua-Ling Wang[1,2], Teng Lei[1],Wen-Qiang Xia[1], Stephen L. Cameron[3], Yin-Quan Liu[1], Zhen Zhang[1], Maruthi M N Gowda[2], Paul De Barro[4], Jesús Navas-Castillo[5], Christopher A Omongo[6], Hélène Delatte[7], Kyeong-Yeoll Lee[8], Mitulkumar V. Patel[2], Renate Krause-Sakate[9], James Ng[10], SanLing Wu[1], Elvira Fiallo-Olivé[5], Shu-Sheng Liu[1], John Colvin[2*], Xiao-Wei Wang[1*]

[1]Insects, Institute of Insect Sciences, Zhejiang University, 866 Yuhangtang Road, Hangzhou 310058, China
[2]Natural Resources Institute, University of Greenwich, Kent ME4 4TB, United Kingdom
[3]Department of Entomology, Purdue University, 901West State Street, West Lafayette, IN 479074, USA
[4]CSIRO EcosystemSciences, Brisbane QLD 4001, Australia
[5]Instituto de Hortofruticultura Subtropical y Mediterránea "La Mayora", Universidad de Málaga – Consejo Superior de Investigaciones Científicas (IHSM-UMA-CSIC), 29750 Algarrobo-Costa, Málaga, Spain
[6]National Crops Resources Research Institute, Namulonge, P.O. Box 7084, Kampala, Uganda
[7]CIRAD, UMR PVBMT CIRAD-Universitéde La Réunion, Pôle de Protection des Plantes, 7 chemin de l'IRAT, 97410 Saint-Pierre, Ile de La Réunion, France
[8]School of Applied Biosciences, Kyungpook National University, Daegu 702-701, Republic of Korea
[9]UNESP, Faculdade de CiênciasAgronômicas, 18610-307, Botucatu, Brazil
[10]Department of Plant Pathology and Microbiology, University of California, Riverside, California 92521, USA

# Correspondence to: John Colvin and Xiao-Wei Wang
Telephone numbers: Colvin = +(44)-(0)1634-883310; Wang =+(86)-(0)571-88982435

Table S1 PacBio sequencing barcode, raw reads and clean reads for each sample.

Sample ID	Barcode1	Barcode2	Raw reads	Clean reads
Asia I	CACACTGAAATCCGTACAGC	ACACGTCACCAGTGTATGCA	3140	2929
Asia II 1	CTAGAGCTGGATCGCAGATC	ATGCTACGTTAGGGCTCGTA	2774	2602
Asia II 3	TCACGACACTAACCTCCGCT	GCTACGATGCACACACGTTA	2368	1921
Asia II 5	GACTAGAGGGATGGTGTTGC	TGCATCGACGGAGCTATGGT	3243	2953
Asia II 6	TACGTACGACACCTGGTGAT	GACAGTGACCTCGTTCGACT	2123	2018
Asia II 7	GCTATACGGACTTTCCCTCG	TCAGTCAGACGTGTACCCAA	3375	3139
Asia II 9	ACACACTGGGAGACAAGGGA	CTACGTAGGTACATACCGGT	2880	2724
Australia	AGACACAGTATCGTTGACCA	CACAGTGTTGAGTCACTGGT	2655	2514
*Bemisia afer*_Africa	AGAGACTGACGCGCAGATAC	CACTCTGTTACAGATGGCTC	3884	3683
*Bemisia afer*_China	TCAGCACAATGTGCACGACT	GACTCTGATCCAAAGTGTTC	3342	3200
China 1	TGACTCAGGCTGATGAGCTG	GTCACTGATCTAGCGTAGTG	3202	3149
China 2	ATATCGCGGAACCAAAGGAT	CGTACGATCACGCCATAATG	3284	3029
Indian Ocean	CGTATACGATCCCGAATTTG	AGAGCTCTAAGGAGCGCCTT	2851	2725
Italy 3	CTAGTGCATCACGGGAGTTG	ACGAGATGACGCCACGAATG	3292	3182
Japan 2	GTAGTGCTCAGTGCATATGC	TCGATCGATCCGAATTCACA	3230	3006
MED	GACACTGTAATCAGTCTCGT	TGACGACTTCCGACACAATT	2866	2820
MEAM1	CACTAGTGTAGGATTGCTCG	ACAGCTCAAAGAGATGTCGA	2831	2710
New World 1	GTACTGCAGTATGCGCTGTA	TCACTCTGCAGGCGTATTGG	4184	3134
New World 2	GCATGCATTTACTGTGCGAT	TAGCTACGGACTTGGTATTC	2860	2696
SubSaharan Africa 1	TGAGCACTGTTGGTCAATCT	GTCAGTGTCGATCCGTATTA	3094	2827
SubSaharan Africa 2	ATCGTACGTAGCTCGTAACT	CTCAGTGAGTCTAATTCCGA	3179	3062
SubSaharan Africa 6	ACAGACAGAGCTGTTGTTTG	CTCACTGTTCGAGGACTGCA	2993	2742
Uganda	CGATGCATAGGCTACACGAC	AGACGTCTTACACGATCTAC	3288	3009

Table S2 PacBio reads and OTUs of bacteria in experimental samples.

OTU ID	New World 1	Asia II 9	Asia I	Asia II 5	Asia II 6	Australia	MEAM1	Bemisia afer_China	Bemisia afer_Africa	China 2	Asia II 7	Italy 3	Japan 2	Indian Ocean	New World 2	MED	SubSaharan Africa 1	SubSaharan Africa 2	Uganda	SubSaharan Africa 6	Asia II 3	Asia II 1	China 1	Consensus Lineage
OTU1	17	3	0	16	1	0	1	0	1	2	0	1	38	0	0	0	0	21	641	21	0	0	5	d__Bacteria; p__Deinococcus-Thermus; c__Deinococci; o__Thermales; f__Thermaceae; g__*Thermus*; s__*Thermus*
OTU2	27	0	0	3	1	0	0	0	0	0	0	2	0	0	0	2	0	1	3	2	0	0	0	d__Bacteria; p__Proteobacteria; c__Alphaproteobacteria; o__Rhizobiales; f__Phyllobacteriaceae; g__*Phyllobacteriaceae*; s__*Phyllobacteriaceae*
OTU3	0	1	0	0	926	0	1785	0	0	0	0	0	0	0	0	0	0	0	0	0	48	0	0	d__Bacteria; p__Proteobacteria; c__Alphaproteobacteria; o__Rickettsiales; f__Rickettsiaceae; g__*Rickettsia*; s__*Rickettsia*_sp.
OTU4	37	1	0	2	1	0	0	0	1	0	0	0	0	0	0	0	0	4	1	1	0	0	1	d__Bacteria; p__Actinobacteria; c__Actinobacteria; o__Propionibacteriales; f__Propionibacteriaceae; g__*Propionibacterium*; s__*Propionibacterium*
OTU5	14	0	0	0	0	0	0	0	0	0	0	0	0	0	0	0	0	0	0	1	0	0	0	d__Bacteria; p__Proteobacteria; c__Alphaproteobacteria; o__Sphingomonadales; f__Sphingomonadaceae; g__*Sphingomonas*; s__*Sphingomonas_panni*
OTU6	46	0	0	19	0	0	1	0	2	0	0	0	3	0	0	4	0	2	5	0	0	0	3	d__Bacteria; p__Actinobacteria; c__Actinobacteria; o__Corynebacteriales; f__Nocardiaceae; g__*Rhodococcus*; s__*Rhodococcus_erythropolis*
OTU7	0	0	0	59	0	0	0	0	0	0	0	0	0	0	0	0	0	0	0	0	0	0	0	d__Bacteria; p__Proteobacteria; c__Gammaproteobacteria; o__Enterobacteriales; f__Enterobacteriaceae; g__*Arsenophonus*; s__*Arsenophonus*
OTU8	47	0	0	0	0	0	0	0	0	0	0	0	0	0	0	0	0	2	0	0	0	0	0	d__Bacteria; p__Proteobacteria; c__Alphaproteobacteria; o__Sphingomonadales; f__Sphingomonadaceae; g__*Sphingomonas*; s__*Sphingomonas_mucosissima*
OTU9	77	2038	1741	1883	191	1211	125	2	1	1793	2149	89	2478	2204	2571	821	919	2173	694	2430	405	1346	97	d__Bacteria; p__Proteobacteria; c__Gammaproteobacteria; o__Oceanospirillales; f__Halomonadaceae; g__*Candidatus*_Portiera; s__*Candidatus*_Portiera_aleyrodidarum_BT-QVLC
OTU10	0	0	360	0	372	1218	0	30	0	941	108	3049	18	0	0	0	1209	346	0	0	1012	0	2689	d__Bacteria; p__Proteobacteria; c__Alphaproteobacteria; o__Rickettsiales; f__Anaplasmataceae; g__*Wolbachia*; s__*Wolbachia*_sp.
OTU11	0	0	0	0	0	0	0	0	0	0	0	0	0	0	0	0	0	0	0	0	70	0	0	d__Bacteria; p__Proteobacteria; c__Gammaproteobacteria; o__Enterobacteriales; f__Enterobacteriaceae; g__*Arsenophonus*; s__*Arsenophonus*
OTU12	0	0	0	0	0	0	0	3155	3467	0	0	0	0	0	0	0	0	0	0	0	0	0	0	d__Bacteria; p__Proteobacteria; c__Gammaproteobacteria; o__Oceanospirillales; f__Halomonadaceae; g__*Candidatus*_Portiera; s__*Candidatus*_Portiera_aleyrodidarum
OTU13	0	65	3	0	54	28	0	0	54	0	40	0	27	0	0	0	0	0	958	0	0	37	0	d__Bacteria; p__Proteobacteria; c__Gammaproteobacteria; o__Enterobacteriales; f__Enterobacteriaceae; g__*Arsenophonus*; s__*Arsenophonus*
OTU14	0	0	1	3	0	0	0	0	0	0	0	0	0	0	0	0	0	0	0	0	0	0	0	d__Bacteria; p__Deinococcus-Thermus; c__Deinococci; o__Deinococcales; f__Deinococcaceae; g__*Deinococcus*; s__*Deinococcus_geothermalis*_DSM_11300
OTU15	97	0	0	26	3	0	0	0	3	2	0	3	3	0	0	7	0	8	11	0	0	0	1	d__Bacteria; p__Proteobacteria; c__Alphaproteobacteria; o__Rhizobiales; f__Hyphomicrobiaceae; g__*Pelagibacterium*; s__uncultured_bacterium
OTU16	0	19	61	33	178	52	0	8	77	0	45	0	81	0	0	0	694	1	463	0	0	60	0	d__Bacteria; p__Proteobacteria; c__Gammaproteobacteria; o__Enterobacteriales; f__Enterobacteriaceae; g__*Arsenophonus*; s__*Arsenophonus_nasoniae*
OTU17	199	0	0	0	0	0	0	0	0	0	0	0	0	0	0	0	0	0	1	0	0	0	0	d__Bacteria; p__Proteobacteria; c__Betaproteobacteria; o__Burkholderiales; f__Oxalobacteraceae; g__*Massilia*; s__*Massilia*
OTU18	0	0	0	28	1	0	1	0	0	0	0	2	170	3	0	0	2	5	15	26	0	0	1	d__Bacteria; p__Proteobacteria; c__Betaproteobacteria; o__Burkholderiales; f__Burkholderiaceae; g__*Cupriavidus*; s__uncultured_bacterium
OTU19	0	0	0	0	0	1	0	0	0	0	784	0	0	0	0	0	0	1	0	0	0	0	0	d__Bacteria; p__Proteobacteria; c__Alphaproteobacteria; o__Rickettsiales; f__Rickettsiaceae; g__*Rickettsia*; s__*Rickettsia*_endosymbiont_of_*Hemiclepsis_marginata*
OTU20	5	0	1	247	0	0	0	0	0	0	0	0	0	0	0	0	0	0	0	0	0	0	0	d__Bacteria; p__Actinobacteria; c__Actinobacteria; o__Micrococcales; f__Microbacteriaceae; g__*Curtobacterium*; s__*Curtobacterium*
OTU21	0	0	0	0	0	0	0	2	34	0	0	0	0	0	0	0	0	0	0	194	0	1114	0	d__Bacteria; p__Proteobacteria; c__Alphaproteobacteria; o__Rickettsiales; f__Anaplasmataceae; g__*Wolbachia*; s__*Wolbachia*_endosymbiont_of_*Bemisia_tabaci*
OTU22	51	0	0	0	0	0	0	0	0	0	0	0	0	0	0	0	0	0	0	5	0	0	0	d__Bacteria; p__Firmicutes; c__Bacilli; o__Bacillales; f__Paenibacillaceae; g__*Paenibacillus*; s__uncultured_Firmicutes_bacterium
OTU23	0	0	0	1	0	0	0	0	0	0	0	0	3	0	0	0	0	0	3	0	0	0	0	d__Bacteria; p__Bacteroidetes; c__Sphingobacteriia; o__Sphingobacteriales; f__env.OPS_17; g__norank; s__unidentified_Cytophagales_OPB73
OTU24	327	0	0	0	0	0	782	0	0	218	0	0	0	0	122	1868	0	0	0	0	80	0	0	d__Bacteria; p__Proteobacteria; c__Gammaproteobacteria; o__Enterobacteriales; f__Enterobacteriaceae; g__*Candidatus*_Hamiltonella; s__*Candidatus*_Hamiltonella_defensa
OTU25	2015	28	12	633	68	4	15	2	43	4	1	35	102	9	0	118	3	151	197	34	2	0	86	d__Bacteria; p__Proteobacteria; c__Gammaproteobacteria; o__Oceanospirillales; f__Halomonadaceae; g__*Halomonas*; s__*Halomonas*
OTU26	150	501	0	0	1	0	0	1	0	0	12	1	83	0	3	0	0	347	0	0	13	0	0	d__Bacteria; p__Bacteroidetes; c__Cytophagia; o__Cytophagales; f__Flammeovirgaceae; g__*Candidatus*_Cardinium; s__*Cardinium*_endosymbiont_of_*Bemisia_tabaci*
OTU27	0	0	750	0	221	0	0	0	0	69	0	0	0	509	0	0	0	0	0	28	291	45	266	d__Bacteria; p__Proteobacteria; c__Alphaproteobacteria; o__Rickettsiales; f__Rickettsiaceae; g__*Orientia*; s__*Rickettsia*_endosymbiont_of_*Bemisia_tabaci*
OTU28	0	68	0	0	0	0	0	0	0	0	0	0	0	0	0	0	0	0	17	0	0	0	0	d__Bacteria; p__Proteobacteria; c__Gammaproteobacteria; o__Enterobacteriales; f__Enterobacteriaceae; g__*Arsenophonus*; s__*Arsenophonus*
OTU29	25	0	0	0	0	0	0	0	0	0	0	0	0	0	0	0	0	0	0	0	0	0	0	d__Bacteria; p__Chlamydiae; c__Chlamydiae; o__Chlamydiales; f__Simkaniaceae; g__*Candidatus* Fritschea; s__*Candidatus* Fritschea bemisiae

Table S3 Comparison of the symbionts found in this and in previous studies.

	A1	A2	A3	A4	A5	C1	C2	F	H	OLO	R1	R2	R3	R4	W1	W2	W3
Asia I				√						√				√	√		
Asia II 1			√	√						√						√	
Asia II 3		√				√			√	√	√				√		
Asia II 5	√			√											√		
Asia II 6			√							√					√		
Asia II 7			√	√		√				√		√			√		
Asia II 9			√	√	√	√											
Australia			√	√											√		
*Bemisia afer*_Africa			√	√												√	
*Bemisia afer*_China				√											√		
China 1				√						√					√		
China 2									√	√					√		
Indian Ocean							√			√							
Italy 3															√		
Japan 2			√	√		√									√		
MED						√			√		√				√	√	
MEAM1									√		√						
New World 1	√						√	√									
New World 2									√								
SubSaharan Africa 1				√			√			√	√				√		√
SubSaharan Africa 2						√									√		
SubSaharan Africa 6																√	
Uganda			√	√	√												

√: represent the strains found by the other studies; √: represent the strains found by this study; √: represent the strains found by this and the other studies.

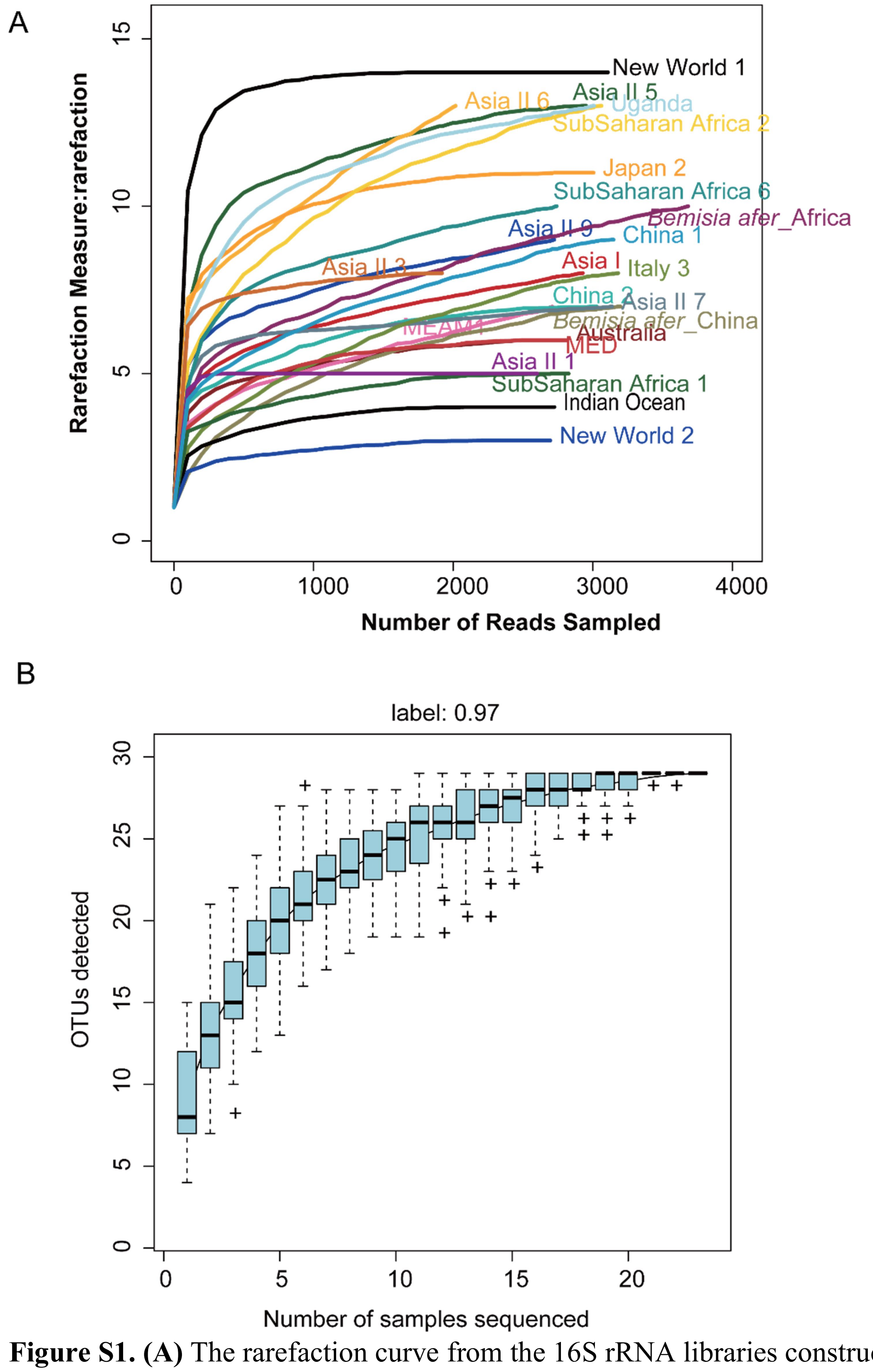

Figure S1. (A) The rarefaction curve from the 16S rRNA libraries constructed for each cryptic species, demonstrating that the amount of sequenced sample was sufficient for most cryptic species. **(B)** Species accumulation curves indicating that total bacteria species richness would probably increase with additional sampling effort.

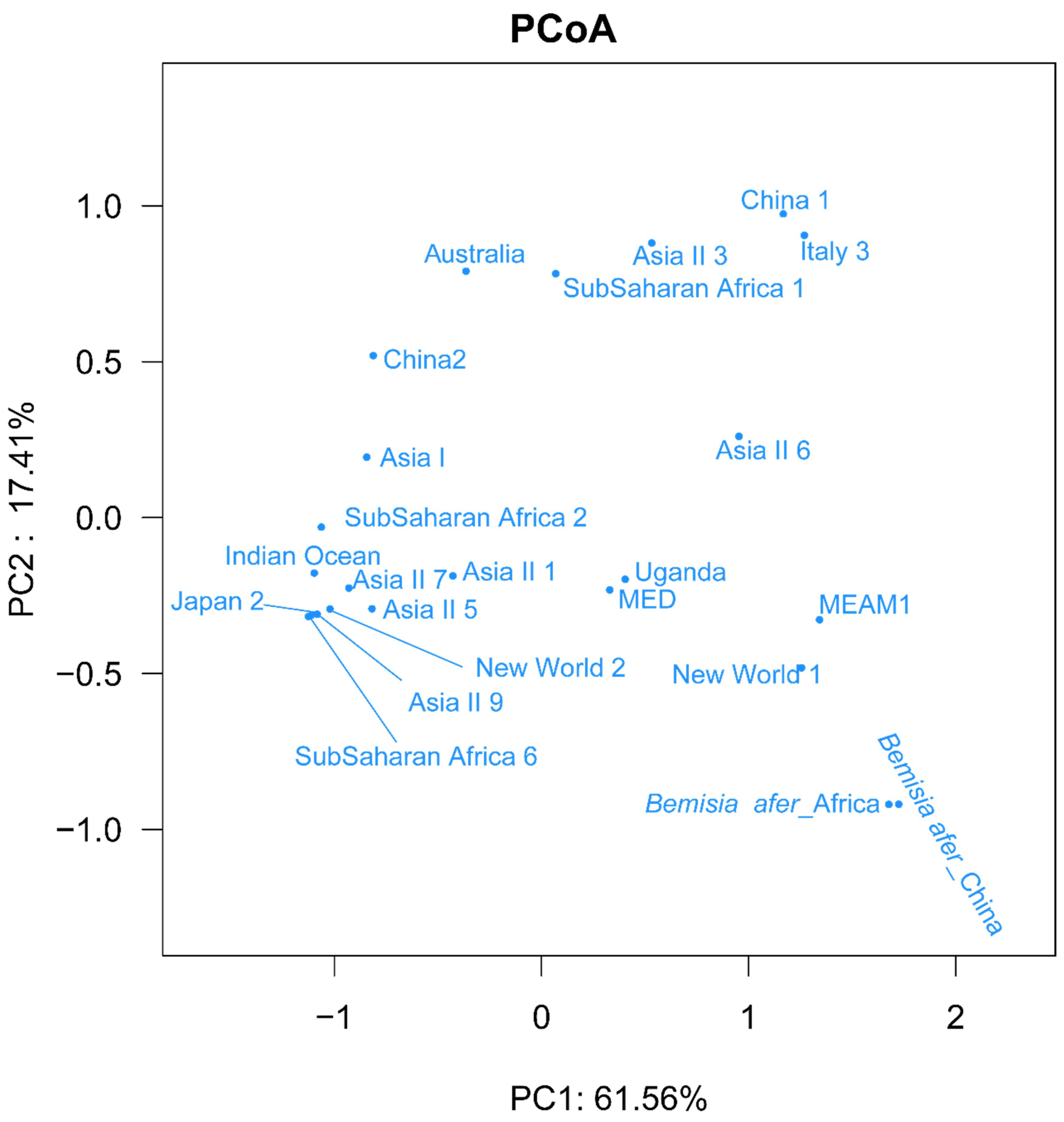

Figure S2. Principal coordinate analysis (PCoA) of unweighted UniFrac distances. Plots were made using Jackknifed UniFrac distances in QIIME (PC1 versus PC2).

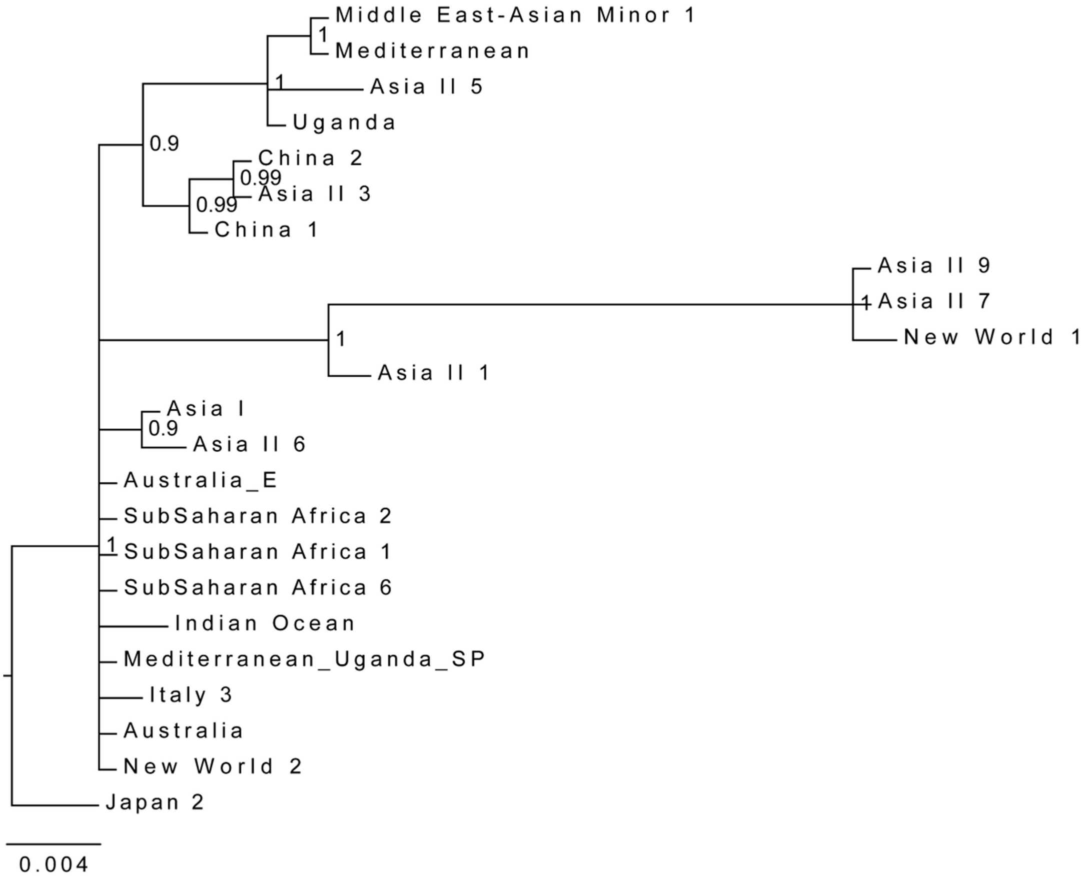

Figure S3. MrBaysian phylogenetic relationships of the *Portiera* from different cryptic species based on 904 bp 16S sequences. BI posterior probabilities were shown on the tree.

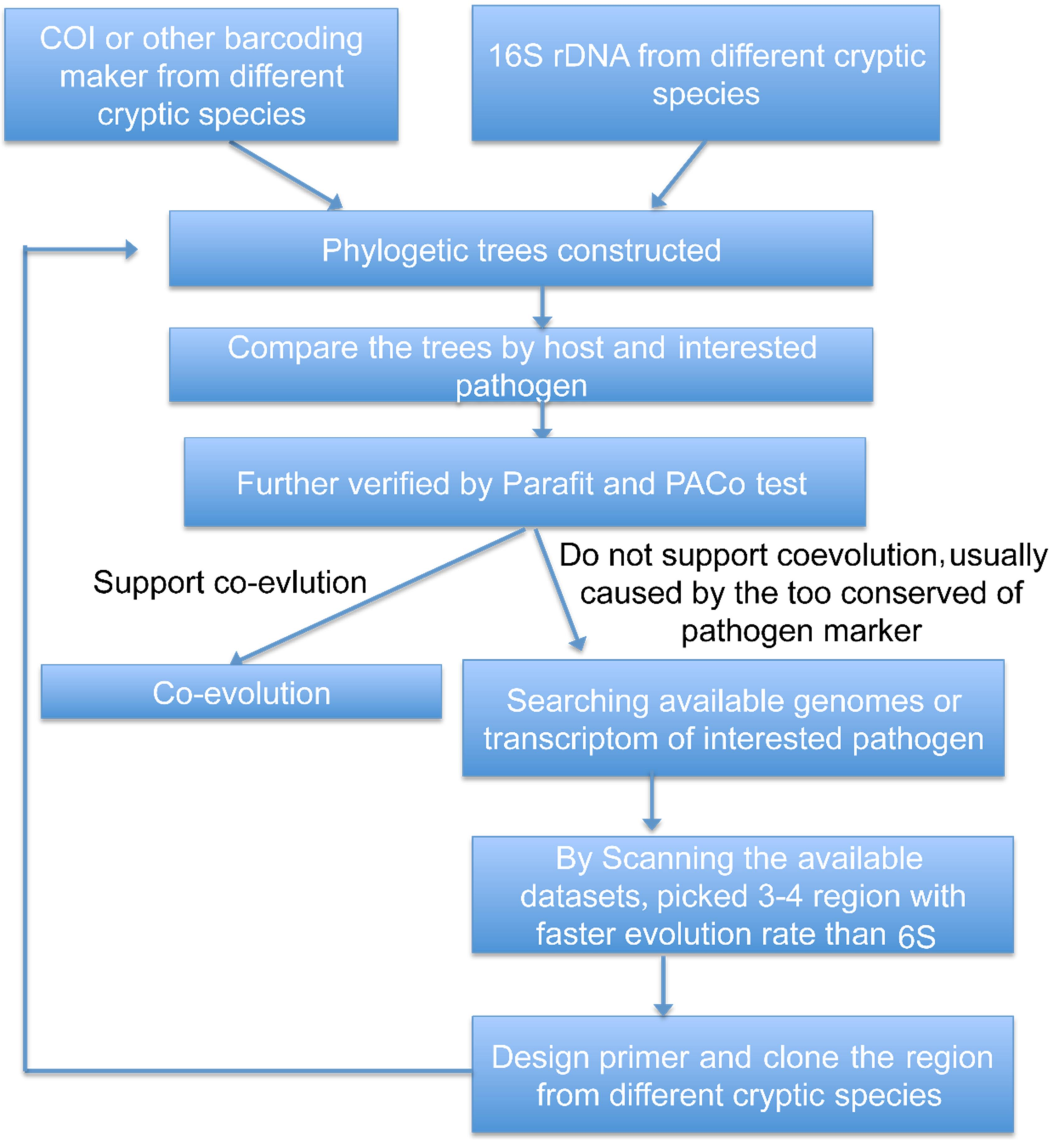

Figure S4. The strategy used to test the hypothesis that phylogenies of P-endosymbionts and their host *B. tabaci* are congruent.
